# The Search for Biomarkers in Hereditary Angioedema

**DOI:** 10.3389/fmed.2017.00206

**Published:** 2017-11-22

**Authors:** Allen P. Kaplan, Coen Maas

**Affiliations:** ^1^Department of Medicine, Medical University of South Carolina, Charleston, SC, United States; ^2^Department of Clinical Chemistry and Haematology, University Medical Center Utrecht, Utrecht University, Utrecht, Netherlands

**Keywords:** angioedema, biomarker, contact system, bradykinin, fibrinolysis

## Abstract

The unpredictable nature of attacks of tissue swelling in hereditary angioedema requires the identification of reliable biomarkers to monitor disease activity as well as response to therapy. At present, one can assess a C4 level (by ELISA) to assist in diagnosis but neither C4 nor C1 inhibitor levels reflect clinical course or prognosis. We will here review a collection of plasma proteins involved in blood coagulation, fibrinolysis, and innate immunity (Figure 1). A main focus is those proteins that are key to the formation of bradykinin (BK); namely, factor XII, plasma prekallikrein/kallikrein, high-molecular weight kininogen, and BK itself since overproduction of BK is key to the disease. Considerations include new approaches to measurement of active enzymes, ELISA methods that may supersede SDS gel analysis of bond cleavages, and examples of changes outside the BK cascade that may reflect when, where, and how an attack of swelling is initiated. We will discuss their usefulness as biomarker candidates, with pros and cons, and compare the analytical methods that are being developed to measure their levels or activity.

## Introduction

Biomarkers are sought because they can be utilized to diagnose, guide therapies, or make predictions regarding the clinical course of the disease in question. Some examples and properties of biomarkers are given in Tables 1 and 2. However, the identification of such markers so that they can be reliably employed, is actually quite difficult. When we consider hereditary angioedema type I or type II (HAE-C1-INH), we can employ C4 as a biomarker because its level is low in 95% of patients when they are asymptomatic (1). Thus, it is a reliable biomarker in patients presenting with recurrent angioedema in the absence of urticaria and is of particular value when a family history is negative or unavailable. The diagnosis is then corroborated with a quantitative and functional determination of C1 esterase inhibitor (C1-INH), which is a diagnostic biomarker that is both more specific than a C4 level and relates directly to the cause of the disease. Yet, the degree of C4 depletion or the functional level of C1-INH in those who have the disease has little relationship to the frequency or severity of attacks and therefore provides little or no information regarding clinical course, prognosis, or even response to therapy. A biomarker that relates to any of these latter three areas would be very helpful.

**Table 1:** Applications and examples of biomarkers.

Usefulness	Example	Analytical method	Reference
Narrows diagnostic possibilities	C4 levels for HAE	ELISA	(2)
Correlates with disease activity	Anti-dsDNA for systemic lupus erythematosus	ELISA	(3)
Has prognostic significance	BRCA1/2 for breast cancer	PCR	(4)

**Table 2:** Properties of an “ideal” biomarker in angioedema.

**Patient management** Offer improved patient diagnosis Ability to stratify patients by severity Assist in therapeutic management (improve patient quality of life)
**Biomarker choice** Easily accessible sample type (e.g., blood) Long half-life in blood Pathobiologic link to disease mechanism
**Assay** Sensitive and specific test Robust assay method (comparable results across multiple labs) Potential for rapid test

**Figure 1:**
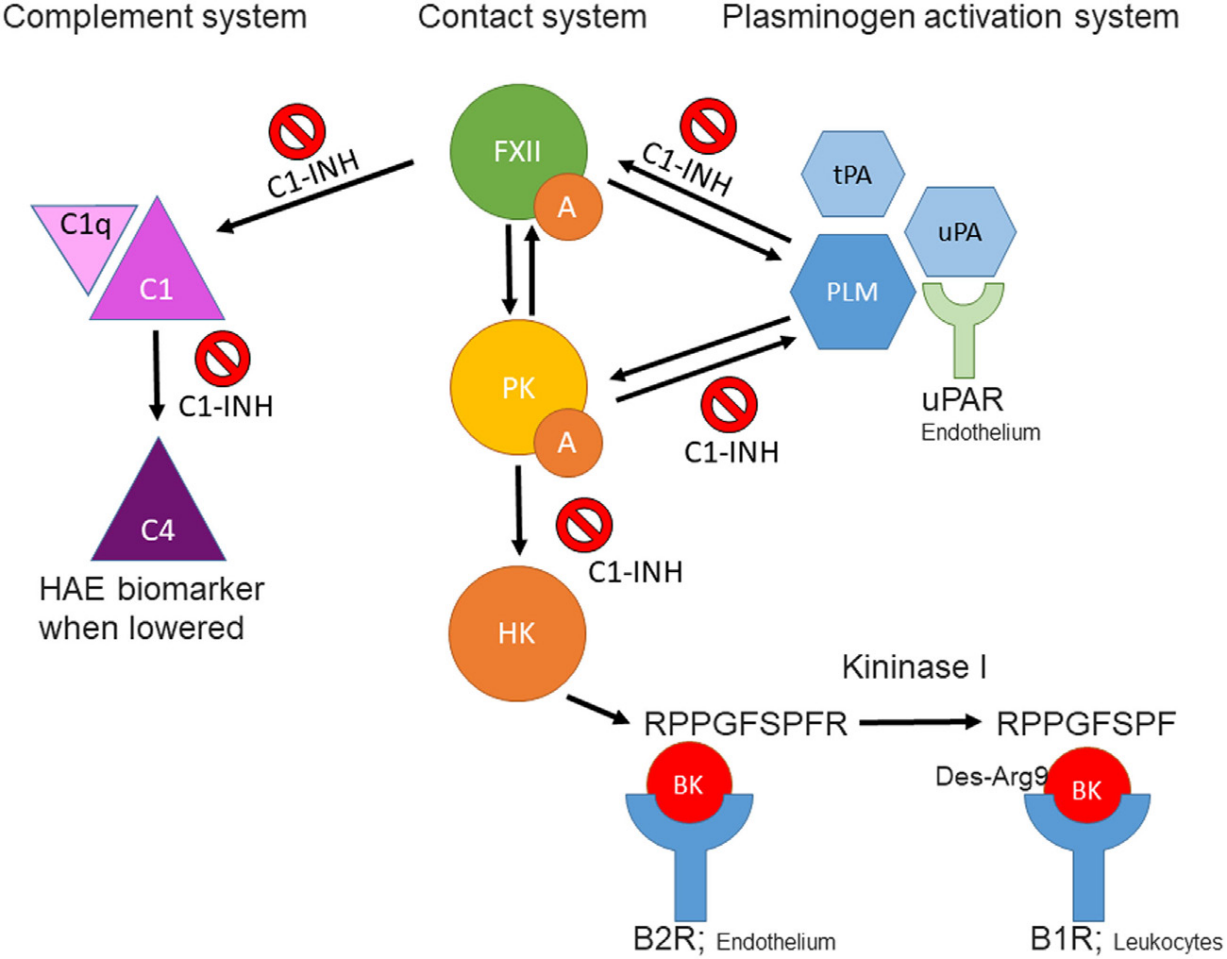
Schematic overview of interactions between the contact-, complement-, and plasminogen-activation systems. FXIIa, activated factor XII; PKa, plasma kallikrein; BK, bradykinin; tPA, tissue plasminogen activator; uPA, urokinase plasminogen activator; PLM, plasmin; C1-INH, C1 esterase inhibitor; B2R, kinin B2 receptor; B1R, kinin B1 receptor; uPAR, urokinase plasminogen activator receptor.

Since bradykinin (BK) is the mediator that causes the swelling (5–8), the BK-forming cascade is a major focus ongoing research hoping that in the course of events, one or more new biomarkers can be identified. Some of these avenues of investigation are considered herein.

## Bradykinin

It is possible to determine (lys-)BK in plasma samples with a competition ELISA or *via* mass spectrometry. This latter technique also enables determination of BK breakdown products (9), which is associated with disease activity in HAE patients (10). While measuring BK directly might seem to be the most obvious (and direct) approach to discern plasma activation and the relationship to symptoms, available methods are particularly lengthy, technically challenging, and very dependent on care in sample collection. Thus, even if done well, it is not practical, and most data reported in the literature are “yes” or “no” in single determinations rather than a profile that can be used to follow a patients’ course. This latter aspect is essential to enable personalized medicine for patients with HAE.

There is a commercial assay kit available for quantitation of a pentapeptide Arg–Pro–Pro–Gly–Phe that is a major product of BK (or lysyl BK) degradation and its utility as a surrogate for BK quantitation might be productive to try; for example, to follow the course of an acute attack of swelling in types I/II HAE. It is produced by a combination of BK degradation due to carboxypeptidases M (cells) (11) or N (plasma) (12), and angiotensin-converting enzyme, or neutral endopeptidase. This approach is confounded by publications that point to aminopeptidase P as an important contributor to BK degradation (13), which would remove the N-terminal Arg and render such a test useless. We disagree with a role for APP in BK degradation (14, 15), and this controversial point should be reexamined using purified constituents to see whether cleavage of the N-terminal Arg–Pro bond is even possible, and then switch to a plasma system. APP does convert lys-BK to BK extremely quickly (16). Much work would be needed using this pentapeptide method to determine whether it could meet any of the biomarker criteria and considerations described earlier.

## High-Molecular Weight Kininogen (HK)

**Figure 2:**
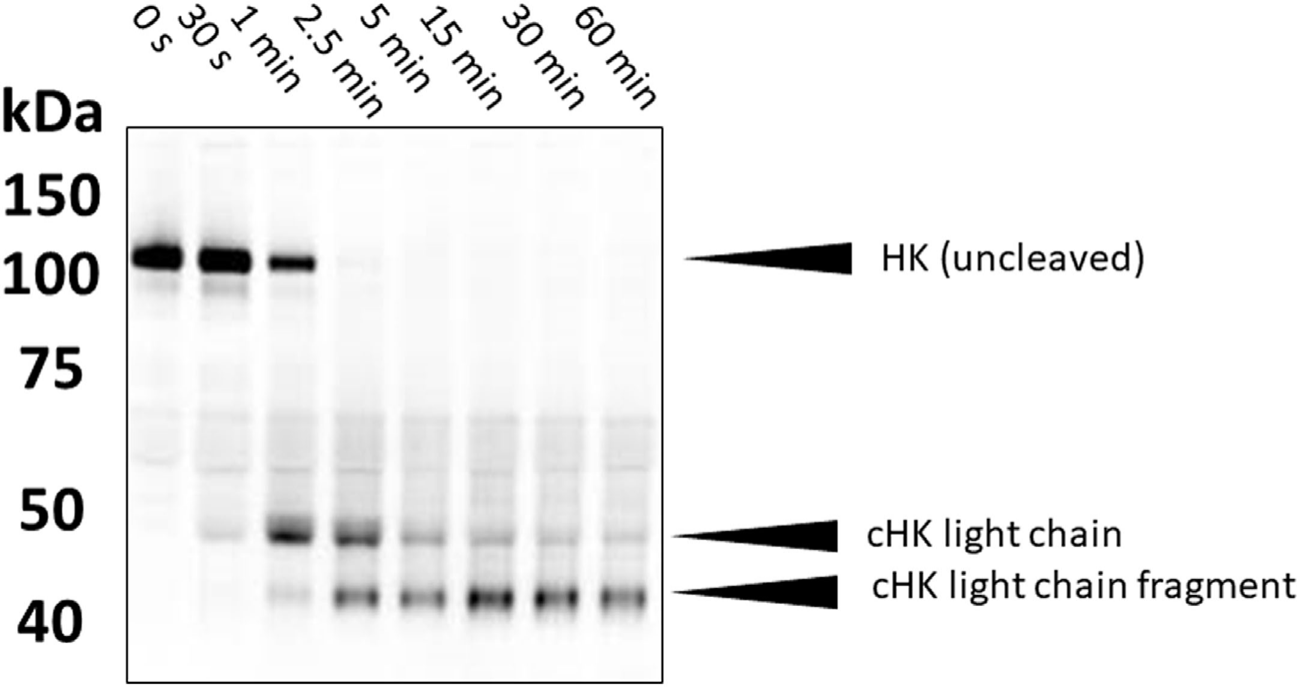
Western blotting of cleaved high-molecular weight kininogen. During contact system activation, single-chain HK is converted into a two-chain disulfide-linked product. In the example shown here, plasma samples (activated *in vitro* with kaolin) were separated on a 4–12% gradient gel under reduced conditions. This separates the two chains. HK was subsequently detected with a polyclonal antibody that preferentially recognizes HK light chain. Molecular weights are shown on the left (in kDa).

High-molecular weight kininogen (HK) is the precursor protein from which BK is enzymatically released. Many methods have been published which seek evidence of cleaved HK as an indirect marker of activation of the BK-forming cascade (17–19). An elevated level can potentially indicate activation of the cascade in a patient with known diagnosis and generally correlates with the presence of angioedema (20). One can examine the mobility shift in cleaved HK vs uncleaved HK in non-reduced SDS gels due to a major conformational change so that it appears smaller even in the presence of SDS. The increment is about 15 kDa (18). Alternatively, one can reduce the sample so that cleaved HK is separated into heavy and light chains, and these can be detected with specific antibody by immunoblot (Figure 2). The exact pattern that is seen depends on the type of antibody (i.e., polyclonal vs monoclonal antibody) and the epitope(s) it reacts with. This is an obstacle in direct comparisons of various studies. Semi-quantitation of HK cleavage can be done by scanning the gels. As a method of quantification, the intensity of expected cleavage products (e.g., cross-reactive with an antibody directed against HK light chain) is compared with the intensity of the remaining uncleaved HK. It should be mentioned that the sensitivity of these assays is variable and hard to standardize; in particular, the HK heavy chain (60 kDa) can become obscured by albumin, which is abundantly present in plasma (66.5 kDa). Analyses of HK cleavage are useful to support therapeutic efficacy of new agents in HAE (21).

The same method can point to a role for the plasma BK-forming cascade when the pathogenesis of any disorder is unclear. More data are needed for other forms of angioedema (i.e., HAE-FXII, HAE-U, and non-histaminergic idiopathic angioedema). Limitations of these approaches include the subjectivity of methods associated with quantitating bands, and background cleavage that is difficult to interpret. This can be due to limitations of sample collection or even plasma instability (activation) *in vivo* that may not relate to clinical manifestations. Some of these problems, particularly ease, speed, and quantitation can be obviated by a new assay for cleaved HK using a monoclonal antibody/ELISA method (22, 23). For all the above assays, it should be noted that although plasma kallikrein (PKa) is a highly effective kininogenase and responsible for BK release, it is certainly not the only enzyme that can cleave kininogen. Detailed studies are needed to pinpoint the exact forms of cleaved HK that are formed during BK production in clinical samples.

## Plasma Kallikrein

When the plasma kinin-forming cascade is activated, PKa activity evolves but there is competition between production and inactivation in terms of plasma levels present. While a monoclonal antibody that recognizes PKa (and not prekallikrein) is in clinical trials, it has not been used to quantitate free PKa, and it is unclear whether that could provide a useful assay (21). PKa binds to C1-INH and α_2_ macroglobulin. Thus there are double antibody ELISA assays for PKa–C1-INH (24) and PKa–α_2_ macroglobulin (25), but the half-life of the complexes is unknown and for HAE types I and II with low functional C1-INH, only the PKa–α_2_ macroglobulin complex could be used to follow an attack of swelling. Whether baseline levels are elevated relative to normal controls needs to be determined as well as its utility to follow the course of attacks of angioedema. Nevertheless, some combination of these methods can be employed along with assays of cleaved HK as described earlier to obtain useful information regarding activation of the plasma BK-forming cascade. As an alternative approach, one can make use of chromogenic or fluorogenic substrates to quantify the overall evolvement of PKa activity in a plasma sample, revealing the overall contribution of factor XII (FXII)-dependent PKa activity and C1-INH activity (26). For quantification, it is possible to use a standard of PKa–α2 macroglobulin complexes. So far, this assay setup requires activation by non-natural triggers (e.g., silica). However, it is imaginable that these can be replaced by more natural, endogenously occurring triggers, once these are definitively identified.

## Plasma Prekallikrein–HK Complex

**Figure 3:**
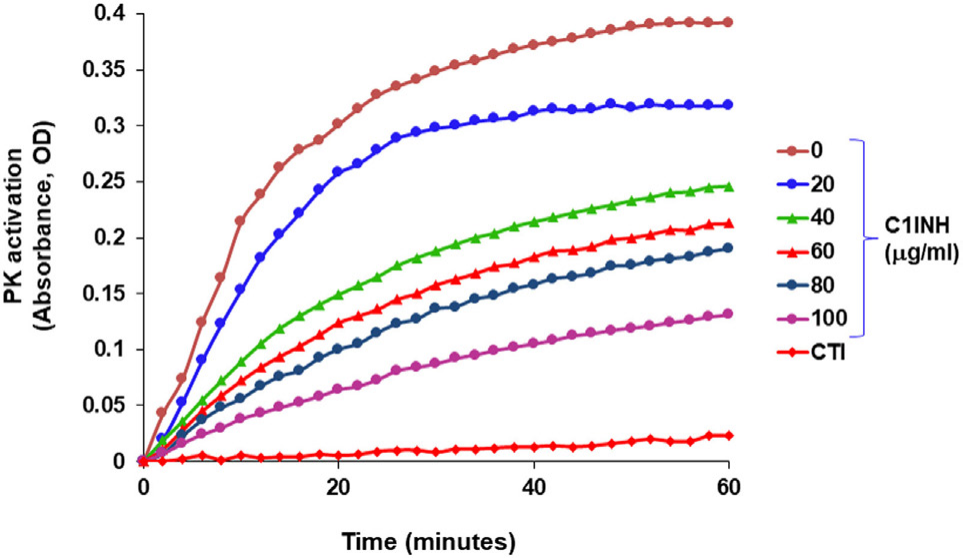
Development of spontaneous plasma kallikrein-like activity in HAE-C1-INH plasma (type I) and the effect of C1-INH and corn trypsin inhibitor (CTI).

**Figure 4:**
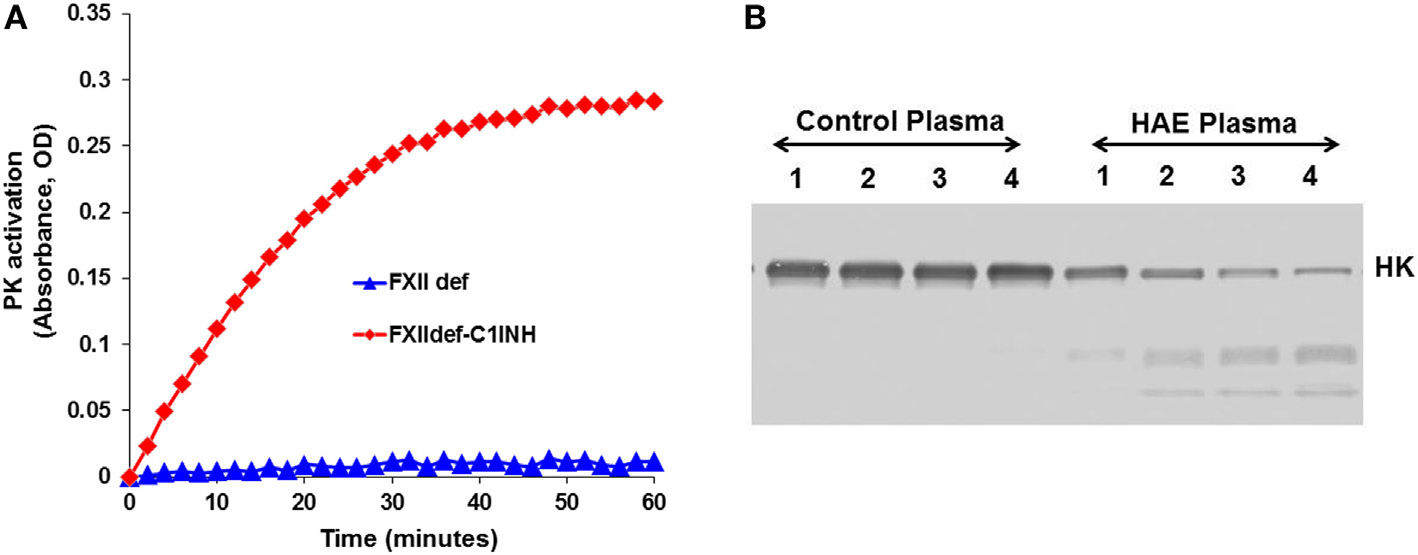
Development of spontaneous plasma kallikrein-like activity in factor XII (FXII)-deficient plasma after depletion of C1-INH. **(A)** Chromogenic substrate conversion assay. **(B)** Western blot for HK cleavage.

In blood plasma, most prekallikrein molecules are non-covalently complexed to HK. This complex already has limited proteolytic activity before prekallikrein activation by molecular scission. This entire process is independent of FXII, and C1-INH can control this process. However, in plasma that is deficient in C1-INH, PKa activity develops spontaneously (27). Previously, we have shown that incubation of plasma of HAE patients in a non-activating plastic test tube at 37°C evolves PKa activity that is not initiated by FXII, but is augmented by it, and is due to autoactivation of the prekallikrein–HK complex in phosphate buffer (28) when C1-INH levels are inadequate. Addition of C1-INH reverses the process (Figure 3). The same result is obtained upon incubation of FXII-deficient plasma (<1%) without an activator if the C1-INH is removed by immunoadsorption (Figure 4A). As C1-INH levels decline below about 70% of normal, there is an inverse dose-dependent rate of PKa formation, and HK is progressively cleaved (Figure 4B). Employing purified prekallikrein and HK, we have shown that the initiating site appears to be within complexed prekallikrein because HK cleavage is stopped by adding corn trypsin inhibitor, which does not inhibit PKa. In non-phosphate buffer, the plasma prekallikrein stiochiometrically cleaves the HK with no PKa formation again pointing to an induced active site within prekallikrein. Prekallikrein uncomplexed to HK is completely stable. This behavior of prekallikrein is very reminiscent of several plasma factors involved in coagulation and fibrinolysis (e.g., FVII, FXII, tissue plasminogen activator, and urokinase plasminogen activator) that are circulating in a “dorment” state and develop limited enzymatic activity during binding to their natural counterparts (tissue factor, an anionic surface, fibrin or urokinase plasminogen activator receptor, respectively). Full elaboration of enzymatic activity follows molecular scission. We consider our assay to determine plasma instability as a result of activity of the prekallikrein–HK complex to represent a biomarker for diagnosis of types I and II HAE (27). In other words, we can diagnose HAE types I/II without measuring C4 or C1-INH. Beyond diagnosis, it cannot be used to predict severity, or clinical course, or impact therapy.

## Factor XII

Factor XIIa–C1-INH complexes can be quantitated when initiation of the plasma BK-forming cascade occurs *via* FXII activation or by activation of FXII by either PKa or plasmin (PLM). Such complexes are elevated during attacks of angioedema in type I or type II HAE (29). This should be interpreted with some caution because of the underlying C1-INH deficiency may influence these results. As a result, it is not a biomarker that can contribute to diagnosis, clinical pattern, therapy choices, or prognosis.

**Figure 5:**
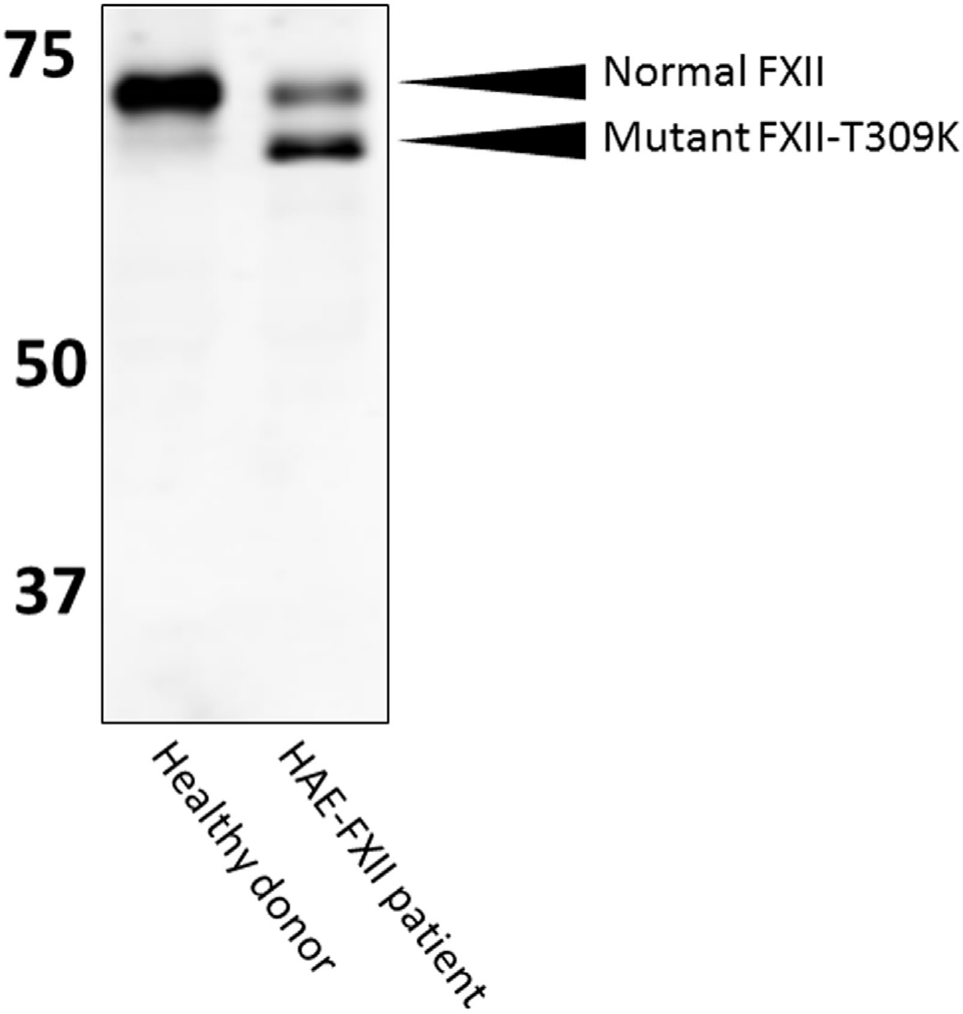
Western blotting of HAE-FXII plasma. Normal plasma contains a single species of factor XII (FXII) protein, but (heterozygous) patients with the HAE-FXII mutation T309K express two forms of FXII. The mutant form lacks an *O*-linked glycosylation, altering its migratory behavior when analyzed by SDS-PAGE.

In HAE-FXII, mutations in the gene that encodes coagulation FXII are reported to cause uncontrolled activity of the plasma contact system, despite the presence of normal levels of C1-INH (30). All these mutations are located in the proline-rich region of FXII that is unique to this protein (31, 32). While we have good evidence for facilitated activation of mutant FXII in HAE-FXII, quantitation of mutant FXIIa-C1-INH during clinical attacks of swelling has so far only been reported in two patients (33). However, it is likely that these complexes form as, the inhibition of mutant FXII by C1-INH appears normal (34). Diagnosis of HAE-FXII requires a method to demonstrate presence of the pathogenic mutations. These mutations are very rare and have so far only been found in a (25%) subset of HAE patients with normal C1-INH levels and activity (35). The minor allele frequency in the 1,000 genomes project (phase 3 combined population) is <0.01 for missense mutations T309K and T309R (www.ensemble.org; rs118204456). However, it should be noted that because a carbohydrate linkage is obviated by the common Lys or Arg substitutions (31) for glycosylated threonine, the molecular weight of the mutant FXII is sufficiently less than normal such that it can be observed in an SDS gel. In heterozygous HAE-FXII patients with T309K or T309R mutations, FXII appears as a doublet, rather than a single band (Figure 5). The lower band represents the pathogenic form of FXII (33, 34). Similarly, it can be expected that a more recently discovered mutation in FXII, that is associated with recurrent angioedema, produces a larger FXII molecule, because of the duplication of a six amino acid sequence (36). In heterozygous patients, this product should be distinguishable on gels with sufficient resolution. In this case, the upper band of the expected doublet (in a heterozygous patient) would represent the suspect pathogenic species. Thus, diagnosis by SDS gel can serve as a diagnostic biomarker for HAE-FXII in the case of the most commonly occurring mutations, and it can be done in one day in the laboratory. Whether this could supplant the current genetic test for the mutation requires confirmation. For other HAE-FXII mutations, a western blotting approach may be less informative: in the case of mutation c.971_1018124del48 (32), 11 amino acids are added to the protein sequence, but 3 target sites for *O*-linked glycosylation are predicted to be eliminated at the same time. As a result, the protein migrates at a height that is indistinguishable from normal FXII (33). Similarly, the molecular weight of mutation A324P is virtually identical to normal FXII and no changes in glycosylation are expected (37), leaving the molecular weight of mutant FXII nearly identical to normal FXII.

## Plasmin and Fibrinolytic Markers

The fibrinolytic system and kallikrein–kinin system are connected at several levels (Figure 1), with implications for our understanding of HAE and other forms of BK-driven vascular leakage. First, C1-INH is a known inhibitor of PLM (38), albeit less potent than α2-antiplasmin. Second, PLM can trigger FXII activation and subsequent activation of the kallikrein–kinin system in the absence of a surface (33, 39). We suppose that this interaction could take place under physiological conditions on the activated endothelium (40), but experimental evidence is needed to confirm this concept. Third, we recently found that three (but not all) HAE-FXII mutations specifically raise the sensitivity of mutant FXII for activation by PLM. This leads to massively increased BK production in plasma, despite the presence of normal levels of C1-INH. It is attractive to hypothesize that a similar mechanism takes place in the recently described new form of HAE with normal C1-INH that is caused by a mutation in the plasminogen gene (41). Together, these findings implicate plasmin as natural contributor to BK production and provide a rationale for the use of the antifibrinolytic agent tranexamic acid as prophylactic therapy in HAE. In similar manner, PLM-mediated activation of the kallikrein–kinin system offers an explanation for the highly dangerous process of brain edema that follows after thrombolytic therapy for stroke (42).

In HAE-C1-INH, elevated levels of PLM–α2 antiplasmin complexes have been reported on several occasions (43–45) indicating an upsurge of PLM activity during swelling episodes. This is accompanied by reports on lowered plasminogen activation inhibitor-1 (PAI-1) levels in HAE-C1-INH (45, 46), as well as in HAE-FXII (47), suggesting that a lowered control of plasminogen activation coincides with swelling attacks. The underlying cause for PLM activity is elusive, as the presence of a preceding injury or intravascular thrombus is not an absolute requirement to develop tissue swelling. Interestingly, the contribution of the fibrinolytic system to HAE extends beyond C1-INH deficiency: in HAE patients with normal C1-INH activity and without FXII mutations (N-HAE), decreased levels of PAI-2 have been documented in a subpopulation of patients under baseline circumstances (48). These determinations of fibrinolytic parameters have provided mechanistic insight into HAE, which may in the future be helpful for selection of an appropriate therapeutic strategy. However, in general, the inter-patient variation in plasma concentrations of these factors appears large, maybe even to an extent that limits their usefulness as biomarkers for personalized medicine.

## Other

While the methods and approaches discussed thus far deal with the core constituents of the plasma BK-forming cascade, there are other areas of research that impact all forms of HAE from which biomarkers might eventuate. C4 is a good example being due to C1r instability when C1-INH is low so that a low level of C1 activation occurs in asymptomatic patients which depletes C4. Yet the level is unrelated to swelling per say.

An area of particular interest is evidence that activation of the lectin pathway could contribute to HAE pathogenesis since mannose-binding lectin-associated serine protease (MASP1) is inhibited by C1-INH (49), and it can be an alternative to PKa as an HK-cleaving enzyme (50). One report indicates that severity may relate to the activity of MASP1, and further studies are needed to determine whether it might be used as a biomarker of course severity and/or prognosis.

Endothelial cell secretion of HSP-90 results in activation of the prekallikrein–HK complex (51) and is a potential source of PKa not only for HK cleavage but also feedback activation of FXII. Its release is stimulated by estrogen, interleukin I, and TNFα. Thus, studies of plasma levels of HSP-90 or one or more of the above cytokines might be of interest regarding possible initiation of attacks of HAE types I and II, as well as HAE-FXII. The same cytokines (but not estrogen) stimulate release of urokinase from endothelial cells (52). Here too, one wonders about a source of PLM for activation of FXII and, in particular mutant FXII in HAE-FXII. Thus assays of urokinase–PAI (1 and 2) complexes, in addition to PLM–α_2_ antiplasmin complexes, might be informative regarding the initiation or course of the disease and if so, given consideration as to possible biomarkers.

Finally, B cell lymphomas express and shed gC1qR (53), thus quantitation of this endothelial cell-binding protein in plasma, in addition to quantitation of C1q levels, might be of interest in assessment of acquired C1-INH deficiency.

## Conclusion

The identification biomarkers and development of accompanying bioassays are urgently needed to accompany clinical trials and enable personalized medicine for HAE. Factors and activation products of the plasma contact system are an important area of scientific interest. Here, technological improvements are needed to increase the sensitivity and clinical applicability of analytical methods. At the same time, new biomarker candidates may present themselves (e.g., from areas like the fibrinolytic system), as we are obtaining increased mechanistic insight into HAE pathology.

## Author Contributions

AK and CM performed literature searches and wrote the manuscript.

## Conflict of Interest Statement

CM is consultant to Shire and Pharming.
